# Global research trends and hotspots of bioresorbable occluders for congenital heart disease: a bibliometric analysis

**DOI:** 10.3389/fcvm.2026.1902660

**Published:** 2026-08-12

**Authors:** Shanshan Li, Yanwen Liu, Chenggang Liu

**Affiliations:** 1 School of Basic Medical Sciences, Heilongjiang University of Chinese Medicine, Harbin, China; 2 Nanjing University of Aeronautics and Astronautics, Nanjing, Jiangsu, China

**Keywords:** bioresorbable occluder, congenital heart disease, endothelialization, patent foramen ovale, transcatheter occlusion

## Abstract

**Objective:**

Bioresorbable occluders have become an important research direction in the interventional treatment of congenital heart disease. Existing reviews have mainly synthesized materials, device designs, and experimental or clinical outcomes, whereas the field's global research structure and temporal evolution remain insufficiently quantified. This study therefore aimed to map research on bioresorbable occluders for congenital heart disease from 2006 to 2026, including its publication trajectory, collaborative structure, knowledge base, thematic evolution, and emerging fronts.

**Methods:**

We searched the Web of Science Core Collection and Scopus databases for related publications from January 2006 to May 2026, limiting document types to articles and reviews and language to English. Bibliometric analysis and visualization were performed using Bibliometrix (R package), VOSviewer, and CiteSpace. Analytic dimensions included annual publication trends, country/region collaboration networks, institutional distribution, journal output, highly cited articles, author collaboration networks, keyword co-occurrence and burst detection, and LDA topic modeling.

**Results:**

A total of 105 publications were included. Annual publication output exhibited marked year-to-year fluctuations, with a local increase during 2009–2012 and renewed, although variable, research activity after 2021; the highest annual output was recorded in 2025 (*n* = 13). Based on affiliation-derived country occurrence counts, China showed the highest representation (266 occurrences) and total citation count (801), followed by the United States and Germany. The United Kingdom, Germany, Singapore, and the Netherlands also demonstrated substantial citation impact. After keyword harmonization, co-occurrence and clustering analyses identified interconnected themes involving congenital defect closure, bioresorbable materials and devices, catheter-based and echocardiography-guided procedures, advanced materials and manufacturing, tissue integration, and clinical outcomes. Burst analysis demonstrated a transition from conventional septal occluders and imaging-guided catheterization toward bioresorbable and percutaneous closure technologies, with bioresorbable, transcatheter closure, bioresorbable occluder and percutaneous closure remaining active through 2026.

**Conclusion:**

Research on bioresorbable occluders for congenital heart disease is accelerating from technical validation toward clinical translation, with China showing prominent representation in the affiliation-derived country distribution and occupying a central position in the international collaboration network. Research attention has expanded from conventional septal occlusion and catheter-based procedures toward bioresorbable materials and devices, degradation and mechanical performance, endothelialization, tissue regeneration, and advanced manufacturing. Recent attention to multicenter evaluation, residual shunt, and migraine further suggests increasing interest in clinical translation and post-closure outcomes. Future research should emphasize the biomechanical coupling mechanisms between degradation kinetics and tissue repair, integrated intelligent functional design, and construction of long-term real-world data risk assessment systems. Deep interdisciplinary integration and global collaborative innovation are expected to drive the vision of intervention without permanent implants and definitive healing toward realization in this field.

## Graphical Abstract

**Figure:**
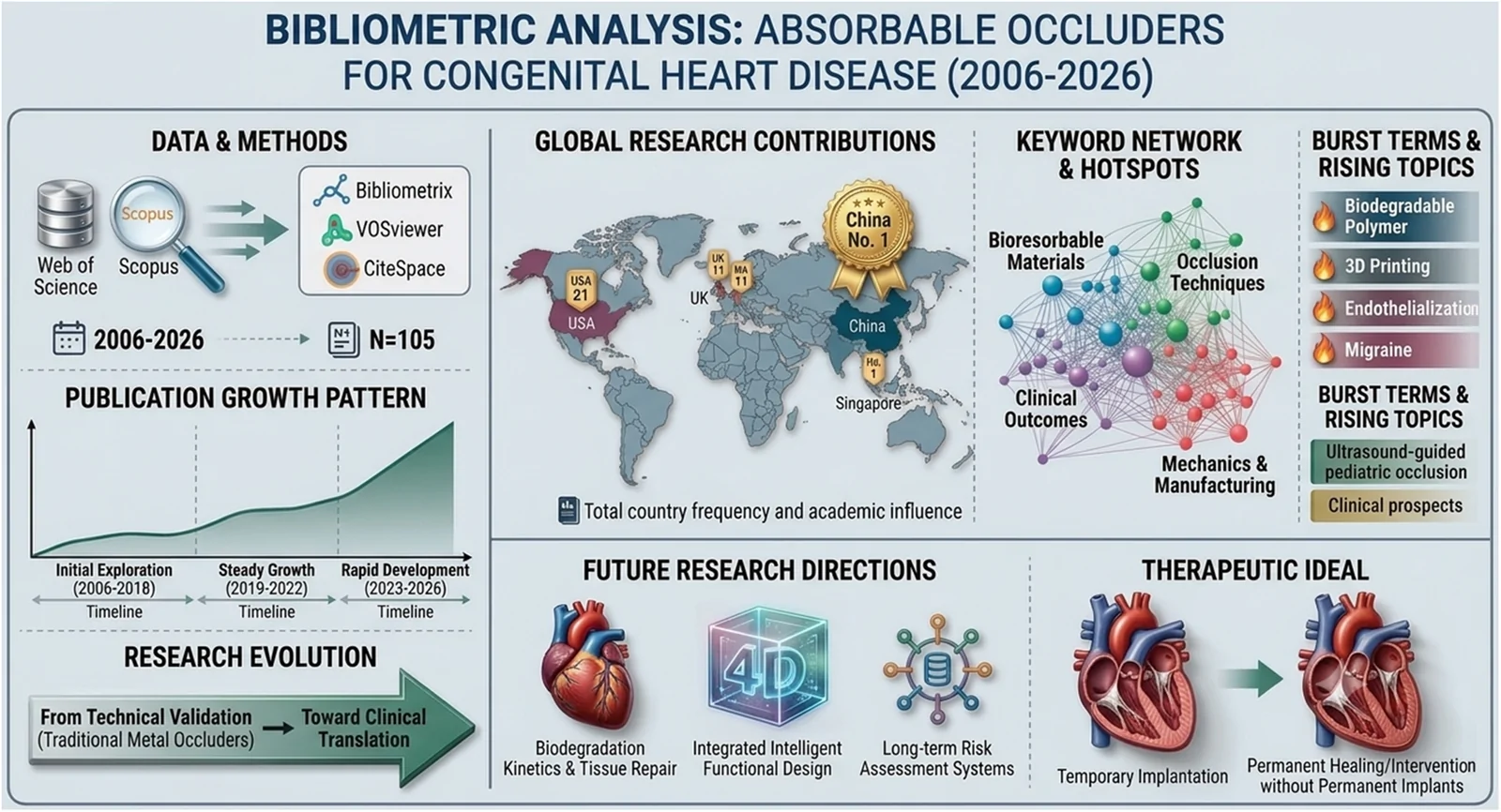

## 1 Introduction

Congenital heart disease (CHD) is the most common birth defect, accounting for approximately one-third of all congenital anomalies (1). Depending on the site of the defect, CHD is mainly classified as ventricular septal defect (VSD), atrial septal defect (ASD), patent ductus arteriosus (PDA), and patent foramen ovale (PFO). VSD has the highest proportion, comprising about 30%–50% (2, 3). Clinical manifestations vary with defect size and location; severe cases may develop heart failure or even pulmonary hypertension. For a long time, open surgical repair was the primary treatment, but it is associated with large trauma, slow recovery, and a high risk of complications. Since the introduction of the Amplatzer occluder, transcatheter interventional occlusion has become the first-line treatment for isolated CHD due to its minimally invasive, safe, and efficient characteristics (4). However, as permanent implants, conventional nitinol occluders present long-term complication challenges. The incidence of complete atrioventricular block after VSD closure ranges from 0.6% to 20%; the core mechanism is persistent mechanical compression of the atrioventricular conduction bundle by the occluder, inducing chronic inflammation and myocardial edema (5). In addition, risks such as cardiac erosion, thrombosis, nickel allergy, and obstruction of future transseptal access are also significant concerns after ASD and PFO closure (6). In PFO patients, paradoxical embolism mechanisms increase the risk of migraine by more than 2.5-fold (7). These issues collectively point to a fundamental conflict: permanently retained metallic foreign bodies vs. the body's physiological requirement for temporary support and permanent healing. The emergence of absorbable occluders offers a breakthrough solution to this dilemma. Their core value lies in the use of biodegradable materials, such as poly-L-lactic acid and its copolymers, which are gradually and completely absorbed by the body after defect closure and tissue repair, serving as a scaffold to guide autologous tissue growth that fills the defect, thereby truly achieving the ideal of intervention without permanent implantation: temporary support and permanent healing (8).

Currently, research in this field is accelerating from passive occlusion toward active repair, with deep integration of intelligent technologies and immune modulation becoming a frontier direction. In the intelligence dimension, near-infrared light-controlled shape-memory occluders can be precisely positioned under ultrasound guidance and remotely activated to deploy, significantly reducing deployment deviation in complex anatomy (9, 10). Meanwhile, 4D printing technology, combining shape-memory polymers with additive manufacturing, can produce personalized occluders that dynamically reconstruct over time, providing a new pathway for precise adaptation and on-demand deformation (11). In the immune modulation dimension, glycocalyx-mimetic hydrogel coatings can promote macrophage polarization toward the reparative M2 phenotype and provide antioxidant protection, signaling a fundamental shift of occluders from physical barriers to bioactive therapeutic platforms (12). Regarding clinical translation, multicenter studies have reported mid-term follow-up results of fully absorbable VSD occluders (13, 14). In addition, expert consensus statements for specific indications such as PFO have been published, indicating that the technology is moving from exploratory stages toward standardized application (15).

Despite significant progress, the field still faces key bottlenecks that limit broad application, including the mechanical stability of biodegradable materials, precise matching of degradation and tissue-regeneration rates, device-related thrombosis risk (16, 17), and applicability in very low-birth-weight infants (18). Existing reviews have provided valuable qualitative syntheses of biodegradable materials, device architectures, preclinical performance, and emerging clinical outcomes (18–20). However, these study-level syntheses were not designed to quantify how the research field is organized or how its contributors, collaboration networks, knowledge foundations, and thematic priorities have changed over time.

Bibliometric analysis is appropriate for these field-level questions because it enables reproducible quantification of publication trajectories, geographical and institutional participation, scientific collaboration, co-citation structures, thematic relationships, and emerging research fronts across a defined literature corpus. It does not replace systematic or narrative reviews, which are better suited to evaluating study quality and synthesizing evidence on the safety and efficacy of individual devices; rather, the approaches are complementary. A previous bibliometric study mapped the much broader patent foramen ovale literature, with major attention to diagnosis, stroke, closure management, and related clinical topics (21). In contrast, the present analysis focuses specifically on bioresorbable occlusion technologies across congenital heart defects and examines their interdisciplinary development at the interface of biomaterials, device engineering, preclinical evaluation, and clinical translation.

Accordingly, this study analyzes literature indexed in the Web of Science Core Collection and Scopus from 2006 to 2026 to characterize the field's temporal development, geographical and institutional distribution, collaborative structure, knowledge base, and evolving research hotspots. By integrating these dimensions, we aim to identify structural gaps and emerging directions relevant to future material innovation, device engineering, and clinical translation.

## 2 Methodology

### 2.1 Data sources and search strategies

**Figure 1:**
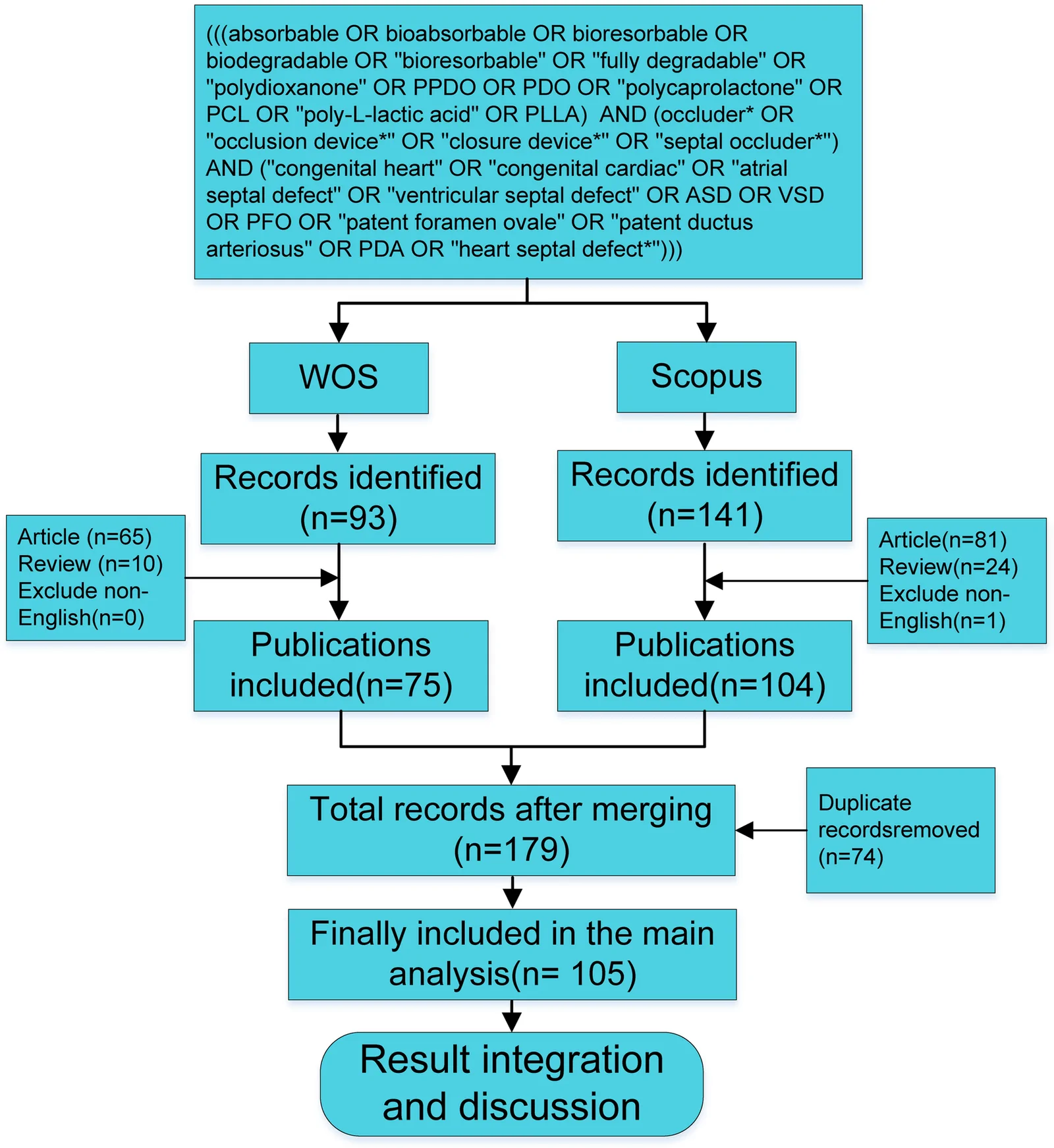
Workflow of the data search strategy.

The data for this study were sourced from two internationally authoritative databases: the Web of Science Core Collection (WoSCC) and Scopus. Searches were completed on 15 May 2026, covering the period from January 2006 to May 2026. Construct a retrieval strategy by combining subject field searching and free-text searching using Boolean logic; the core search string is provided in the Supplementary Material. Applying the above search string in the Web of Science Core Collection yielded 93 records. Limiting document types to Article and Review and language to English resulted in 75 records. The same search strategy was executed in the Scopus database, initially retrieving 141 records; after applying identical document type and language limits, 104 records remained. The two databases together produced 179 records. These 179 records were imported into reference management software and duplicates were removed by comparing titles, authors, journals, publication years, and DOI information, eliminating 74 duplicate records and yielding a final set of 105 documents for the main analysis. The detailed screening process is shown in Figure 1. Subsequent bibliometric analyses, synthesis of results, and discussion were based on these 105 documents.

### 2.2 Bibliometric analysis

Three bibliometric software tools were used for data analysis and visualization. Before all keyword analyses, author keywords (DE) and Keywords Plus (ID) were harmonized using a prespecified label/replace-by thesaurus. The standardization process addressed differences in capitalization, whitespace, Unicode hyphens, singular and plural forms, British and American spelling, abbreviations and full terms, and device-level synonyms. ASD, VSD, PFO, PDA, and CHD were expanded to their canonical full forms. Device-level descriptors containing biodegradable, bioabsorbable, absorbable, or bioresorbable were consolidated under bioresorbable, whereas materially or clinically distinct concepts—including biodegradable polymer, specific polymer names, adult congenital heart disease, and lesion-specific terms—were retained separately. Congenital heart defect(s) was mapped to congenital heart disease only when it was used as a general umbrella term. Incomplete or non-thematic indexing terms were excluded, and duplicate canonical terms within the same document were counted only once. The final thesaurus comprised 109 auditable label-level decisions, including 34 exclusions.

The combined DE and ID terms were used for the all-keyword word cloud, timeline, burst, trend-topic, and CiteSpace analyses, whereas DE terms alone were used for the author-keyword word cloud and co-occurrence network. VOSviewer (version 1.6.20) was used to construct the author-keyword co-occurrence network, with a minimum occurrence threshold of two documents. CiteSpace (version 6.4.R1) was used for keyword burst detection and cluster visualization. The time-slice length was set to one year, and nodes were selected using the g-index (*k* = 25); the remaining parameters were LRF = 2.5, L/N = 10, LBY = 5, and *e* = 1.0. Bibliometrix/Biblioshiny was used to calculate keyword frequencies and generate the word clouds, timeline map, and trend-topic analysis.

Author and institutional name normalization, collaboration network construction, co-citation analysis, annual publication analysis, and LDA topic modeling were performed using the procedures detailed in the Supplementary Material. The Supplementary Material provide the complete preprocessing workflow, software-specific parameter settings, and model validation procedures.

## 3 Results

### 3.1 Publication trends and overview of research output

**Figure 2:**
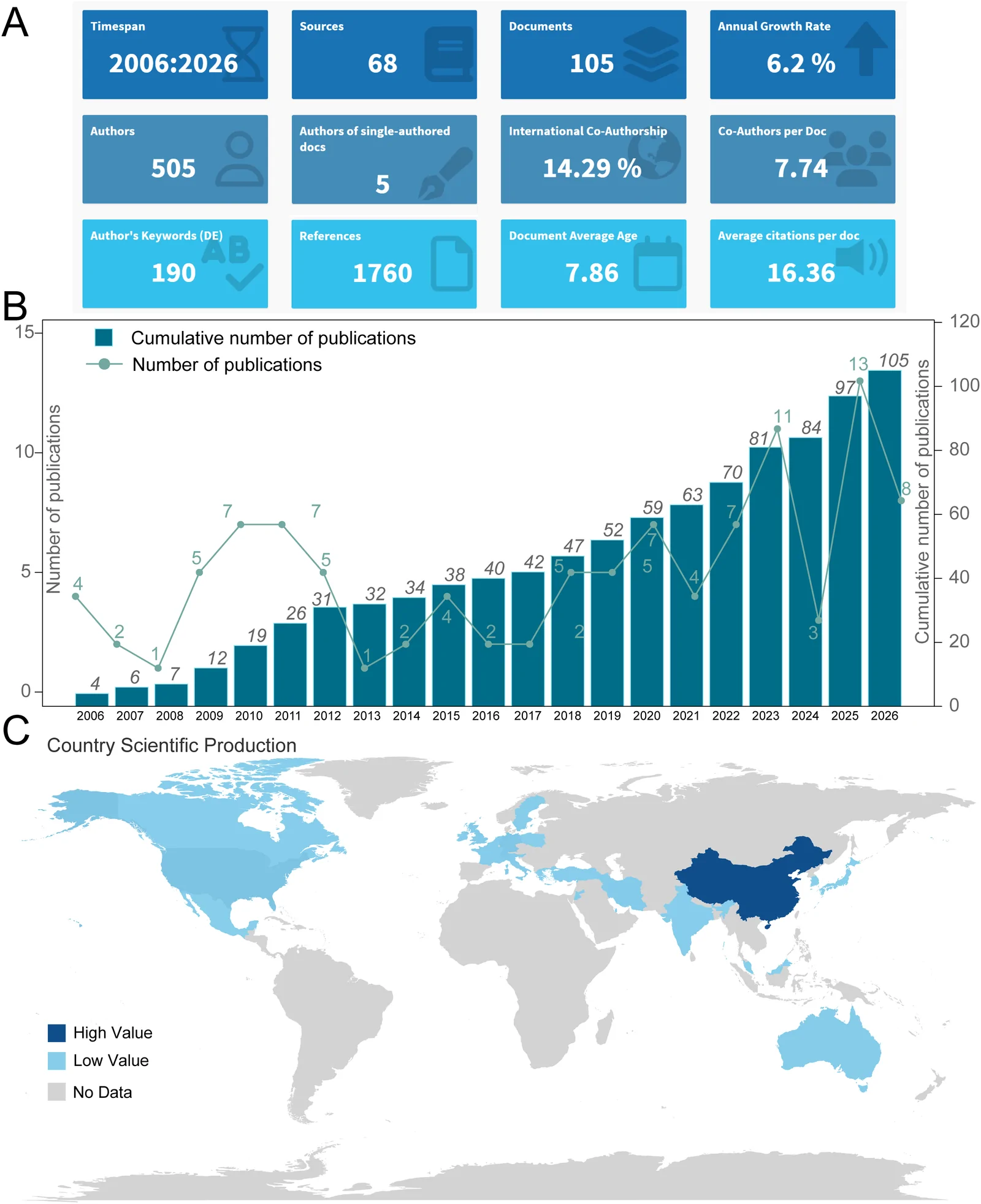
(**A**) Overview of basic information; (**B**) annual publication trend; (**C**) map of national scientific output.

This study included 105 publications spanning 2006 to 2026, comprising 84 Articles and 21 Reviews (Supplementary Table 1; Figure 2A). As shown in Figure 2B, annual publication output exhibited substantial year-to-year fluctuations rather than a sustained pattern of continuous growth. A local increase was observed between 2009 and 2012, with 5, 7, 7, and 5 publications, respectively. Annual output subsequently fluctuated between 1 and 7 publications from 2013 to 2021. Research activity increased again after 2021, although the annual counts remained variable, with 7, 11, 3, 13, and 8 publications recorded from 2022 to 2026, respectively. The 2026 count represents partial-year data and should therefore not be directly compared with counts for complete calendar years. Overall, the cumulative number of publications increased from 4 in 2006 to 105 in 2026. In terms of country frequency (Figure 2C), Asia (particularly China), Europe, and North America are the most represented regions, indicating their leading roles in collaborative research output.

### 3.2 International collaboration patterns and major contributing countries

**Figure 3:**
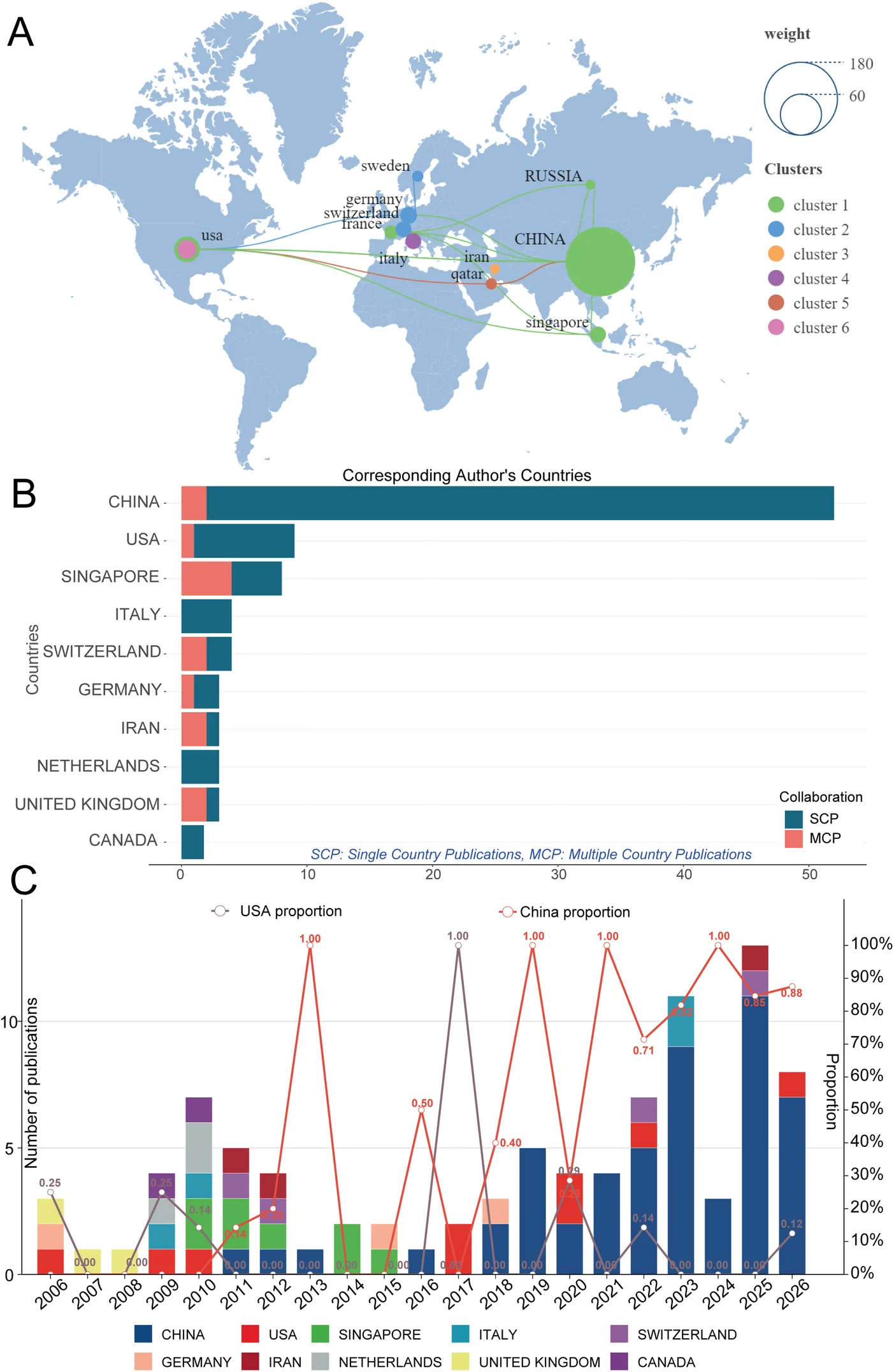
(**A**) Country collaboration network map; (**B**) distribution of publications by country of corresponding author; (**C**) temporal trends of research output for major countries.

Figure 3A depicts the intercountry research collaboration network. CHINA is the unequivocal core node, having established extensive collaborations with the United States, Singapore, Germany, Switzerland, France, and others, forming a China-centered star-shaped collaboration pattern. Russia, Italy, Iran, Qatar, and other countries also participate in collaborations of a certain scale. The distribution map of corresponding authors’ countries in Figure 3B further confirms China's leading position. The trend of national output over time in Figure 3C shows that after China broke through from zero in 2011, its publication count grew exponentially and is projected to reach 52 papers in 2026; the United States, Italy, and Switzerland exhibit relatively modest growth, while Singapore experienced rapid growth from 2010 to 2015 before entering a plateau.

Table 1 presents the ten countries/regions with the highest affiliation-derived occurrence counts. China showed the highest representation, with 266 affiliation occurrences and 801 total citations, followed by the United States (23 occurrences) and Germany (18). Singapore and Switzerland each recorded 16 occurrences. Although the United Kingdom had only nine affiliation occurrences, its publications accumulated 168 citations. These results indicate substantial representation of China in the author-affiliation data; however, the occurrence count should not be interpreted as the number of unique publications produced by a country.

**Table 1:** Top 10 countries/regions by country frequency.

Country	Frequency	Time cited
CHINA	266	801
USA	23	44
GERMANY	18	123
SINGAPORE	16	173
SWITZERLAND	16	31
UK	9	168
IRAN	7	12
ITALY	7	25
FRANCE	5	7
NETHERLANDS	5	65

Country occurrences were derived from country information in the complete author-affiliation data. Because multiple affiliation-linked country instances may be recorded for a single publication, occurrence counts can exceed the total number of included publications (*N* = 105). These values reflect representation in the affiliation data rather than the number of unique publications attributable to each country.

### 3.3 Institutional and funding support analysis

**Figure 4:**
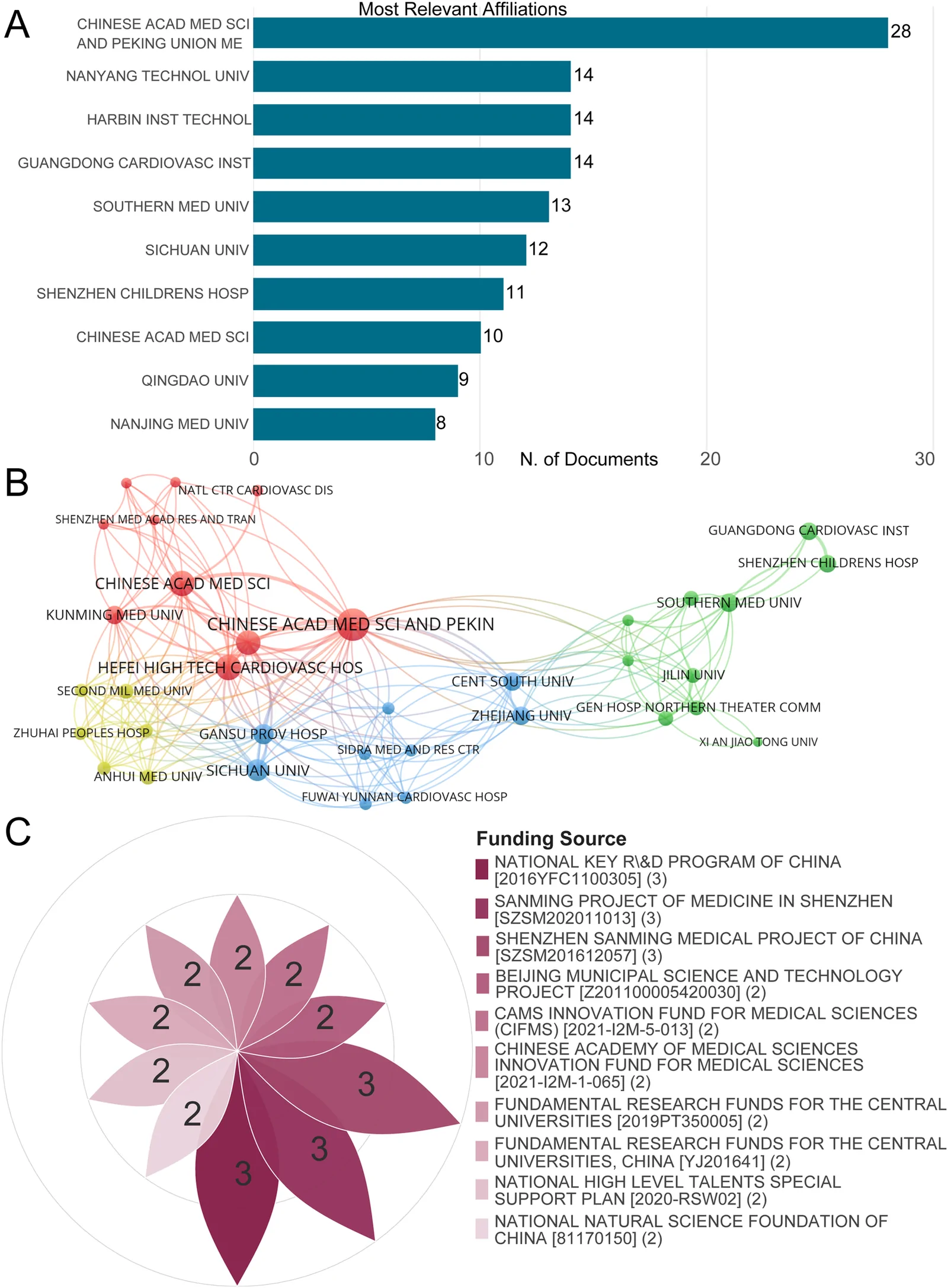
(**A**) High-output institutions by publication count; (**B**) institutional collaboration network; (**C**) distribution of major funding sources.

Figure 4A shows the top five institutions by publication count: Chinese Academy of Medical Sciences & Peking Union Medical College, Nanyang Technological University, Harbin Institute of Technology, Guangdong Cardiovascular Institute, and Southern Medical University. The Chinese Academy of Medical Sciences system leads in publication volume; Nanyang Technological University is the only international institution among the top 10. The collaboration network in Figure 4B forms four clusters. Cluster 1 is centered on the Chinese Academy of Medical Sciences and Peking Union Medical College, connecting institutions such as Hefei High Tech Cardiovascular Hospital and Nanjing Medical University; Cluster 2 is centered on Guangdong Cardiovascular Institute and Shenzhen Children's Hospital, radiating across South China; Cluster 3 includes Sichuan University, Central South University, and Zhejiang University; Cluster 4 contains Anhui Medical University, Second Military Medical University, and Shenzhen Medical Academy of Research and Translation. Second Military Medical University has the earliest mean publication year (2016), while Anhui Medical University and others are more recent (2025). Figure 4C shows the main funding sources are the National Key R&D Program of China (3 projects), Sanming Project of Medicine in Shenzhen (3 projects), CAMS Innovation Fund (2 projects), and NSFC (2 projects), indicating strong national and local financial support.

### 3.4 Analysis of journal output and impact

**Figure 5:**
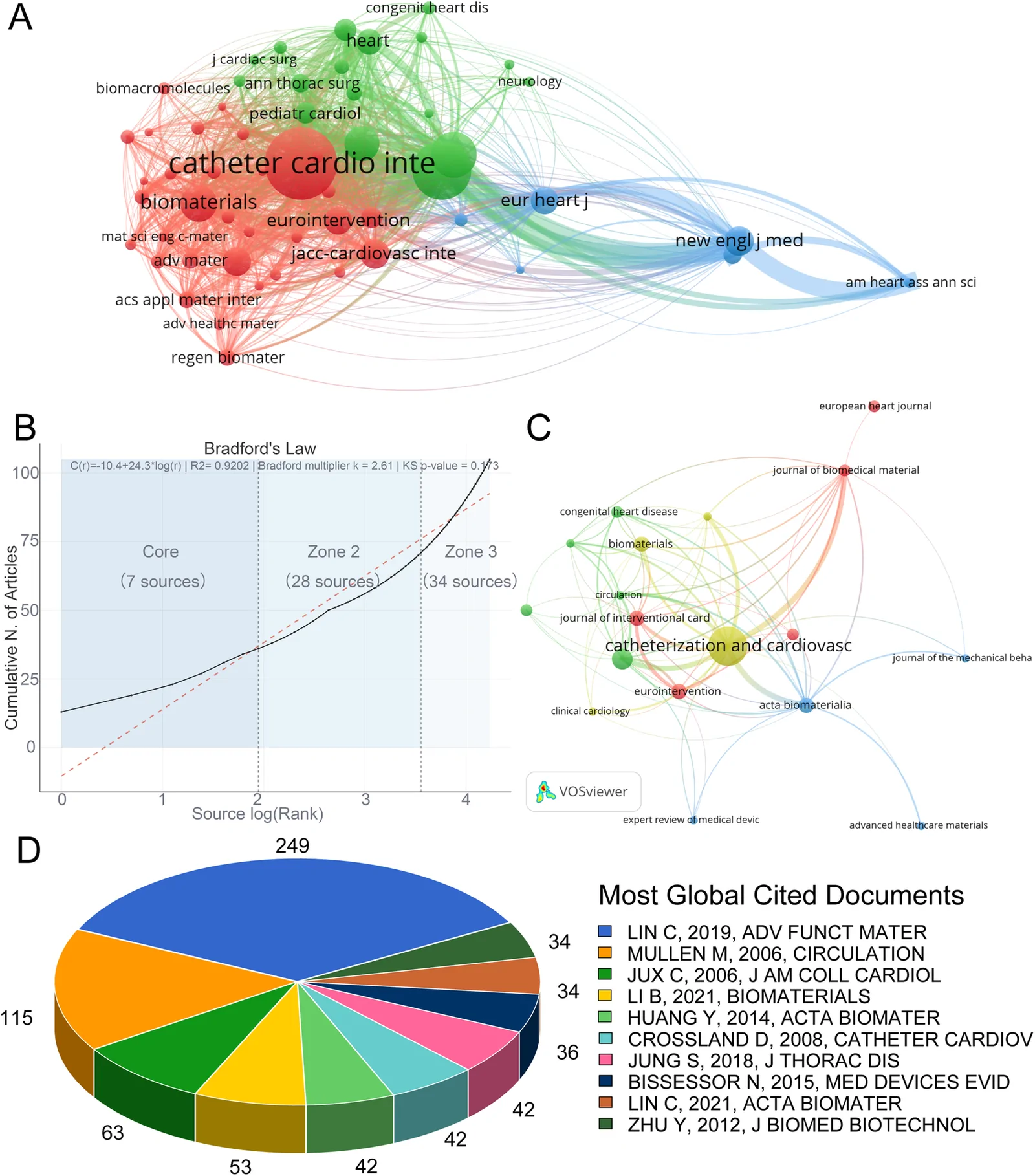
(**A**) Journal citation network; (**B**) Bradford's law; (**C**) journal co-citation network; (**D**) journal publication volume pie chart.

Table 2 presents the journals with the highest publication counts. Catheterization and Cardiovascular Interventions ranks first with 13 papers, followed by Frontiers in Cardiovascular Medicine, Acta Biomaterialia, and Biomaterials. By impact factor, Circulation, Biomaterials, and Acta Biomaterialia occupy the top three positions. The pie chart in Figure 5D shows that the top six journals by publication volume constitute the core zone, contributing nearly one-third of all documents. The Bradford zone plot in Figure 5B further confirms the distribution of these core journals, consistent with the typical Bradford's law (Supplementary Table 2). Both the journal co-citation network (Figure 5C) and the journal citation network (Figure 5A) reveal four clusters of journals: interventional cardiology (red), general cardiovascular journals (green), biomaterials and biomechanics (blue), and the intersection of materials and intervention (yellow). The yellow cluster has the densest connections with the red and blue clusters, indicating that research on absorbable occluders deeply integrates material degradation behavior, mechanical properties, and interventional surgical techniques; interdisciplinary collaboration is a core driving force for development in this field.

**Table 2:** High-impact journals.

Publisher	Journal name	H_index	TC	NP	Journal citation reports quartile	Impact factor	PY_start
WILEY	CATHETERIZATION AND CARDIOVASCULAR INTERVENTIONS	10	233	13	Q3	1.9	2008
ELSEVIER	ACTA BIOMATERIALIA	4	130	4	Q1	9.6	2010
ELSEVIER	BIOMATERIALS	4	105	4	Q1	12.9	2011
FRONTIERS	FRONTIERS IN CARDIOVASCULAR MEDICINE	3	28	6	Q3	2.9	2022
WILEY	JOURNAL OF INTERVENTIONAL CARDIOLOGY	3	43	4	Q3	1.7	2018
SPRINGER	PEDIATRIC CARDIOLOGY	3	23	3	Q4	1.4	2016
Lippincott	CIRCULATION	2	118	2	Q1	38.6	2006
Europa Group	EUROINTERVENTION	2	50	2	Q1	9.5	2010
TAYLOR & FRANCIS	EXPERT REVIEW OF MEDICAL DEVICES	2	17	2	Q3	2.7	2007
WILEY	JOURNAL OF BIOMEDICAL MATERIALS RESEARCH PART B-APPLIED BIOMATERIALS	2	15	2	Q3	3.4	2018

Table 3 lists the top ten locally cited documents. JUX C (2006) was cited locally 29 times, accounting for 46.03%; LIN C (2021) was cited locally 14 times, accounting for 41.18%; SIGLER M (2018) was cited locally 12 times, accounting for 42.86%. The local citation proportions for KARAGIANNI A (2011) and SONG L (2022) reach 54.55% and 55.56%, respectively, demonstrating their distinctive influence within the field. LIN C (2019) has a total of 249 citations, but only 5 local citations (2.01%), indicating a prominent cross-disciplinary impact.

**Table 3:** Ranking of highly cited documents in the field.

Document	DOI	LC	GC	LC/GC Ratio (%)
JUX C, 2006, J AM COLL CARDIOL	10.1016/j.jacc.2006.02.057	29	63	46.03
LIN C, 2021, ACTA BIOMATER	10.1016/j.actbio.2021.04.050	14	34	41.18
SIGLER M, 2018, EUROINTERVENTION	10.4244/EIJ-D-17-00006	12	28	42.86
KARAGIANNI A, 2011, SCAND CARDIOVASC J	10.3109/14017431.2011.591819	6	11	54.55
LIN C, 2019, ADV FUNCT MATER	10.1002/adfm.201906569	5	249	2.01
SONG L, 2022, FRONT CARDIOVASC MED	10.3389/fcvm.2022.945275	5	9	55.56
MATSUZAKI Y, 2022, PEDIATR CARDIOL	10.1007/s00246-021-02809-5	3	6	50
CALLEGARI A, 2022, J INTERV CARDIOL	10.1155/2022/3476398	2	5	40
CONG J, 2024, FRONT CARDIOVASC MED	10.3389/fcvm.2024.1420704	2	4	50
LIN C, 2023, ADV HEALTHC MATER	10.1002/adhm.202201999	1	18	5.56

### 3.5 Core author analysis

Table 4 lists the top ten authors by publication count. Zhang Z ranked first with 29 publications, followed by Wang S with 14; Li B, Pan X, and Xie Y each published 10 papers. By institutional distribution, Guangdong Cardiovascular Institute contributed the largest number of high-output authors; Fuwai Hospital and Shenzhen Children's Hospital are also represented. Zhang Z has the highest H-index (24), indicating notable academic impact.

**Table 4:** High-output author rankings.

Author	Articles	Country	H-index	Affilliations	PY_start
ZHANG Z	29	CHINA	24	Guangdong Cardiovascular Institute	2016
WANG S	14	CHINA	14	Guangdong Cardiovascular Institute	2016
LI B	10	CHINA	4	Guangdong Cardiovascular Institute	2019
PAN X	10	CHINA	5	Guangdong Cardiovascular Institute	2018
XIE Y	10	CHINA	7	Shenzhen Children's Hospital	2019
LI Y	9	CHINA	4	Guangdong Cardiovascular Institute	2019
WANG Y	9	CHINA	5	Fuwai Hospital	2022
CHEN J	8	CHINA	6	Southern Medical University, Guangzhou	2011
WANG W	8	CHINA	7	Nankai University	2018
LI Z	7	CHINA	6	Fuwai Hospital	2012

**Figure 6:**
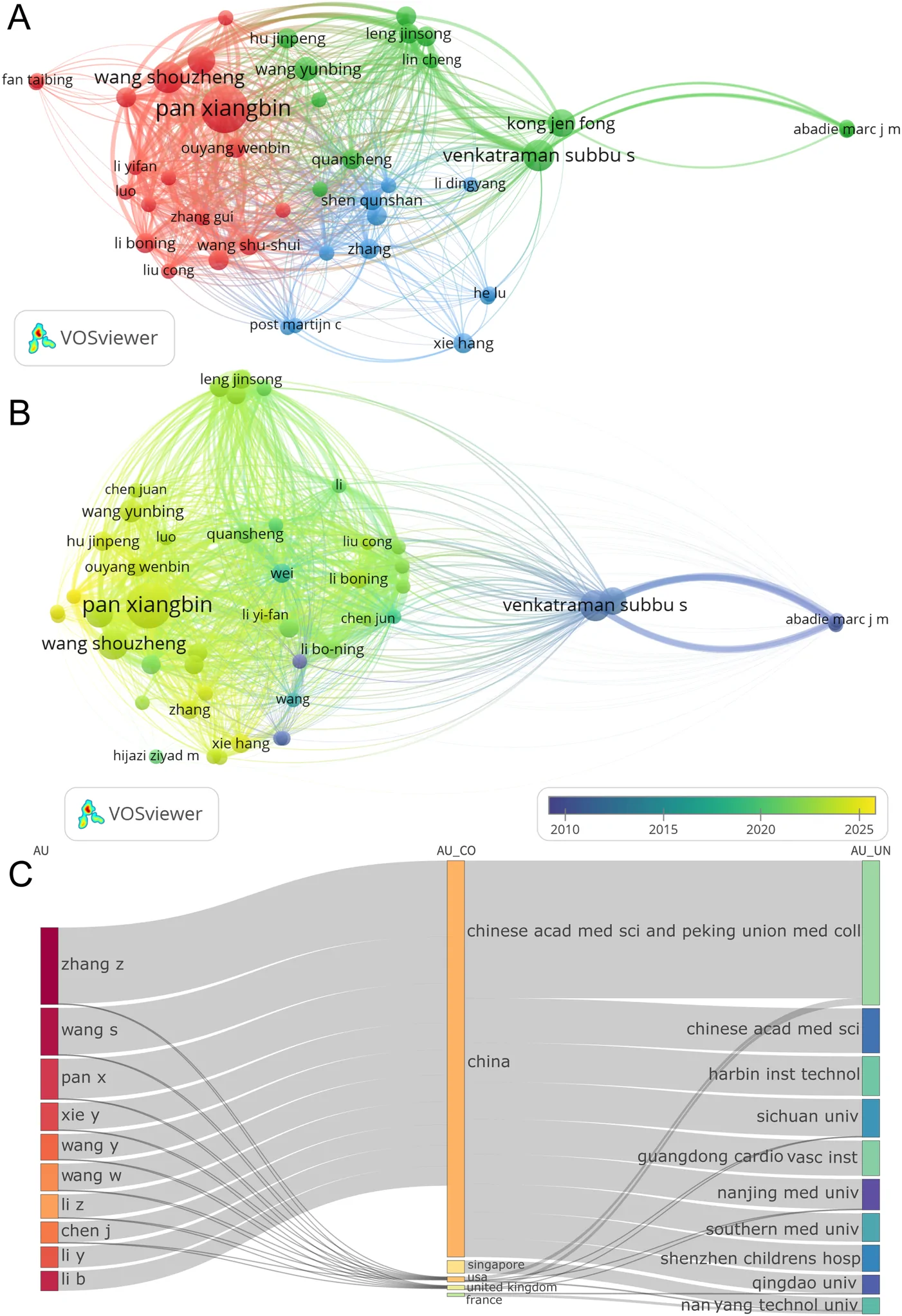
(**A**) Author co-citation network (3); (**B**) author bibliographic coupling network (3); (**C**) author–country–institution three-field plot.

Figure 6A formed five clusters. The red cluster (Pan X, Wang S, Zhang F, Hu J) represents the most active team in recent years; the blue cluster (Kong J, Leng J, Lin C) includes early foundational authors; the green cluster (He L, Post M, Xie H) is dominated by international collaborators. The structure of Figure 6B is similar to Figure 6A, with Pan X, Wang S, and Zhang F still occupying central positions. New authors such as Chen J and Ouyang W are beginning to emerge. In Figure 6C, the connections on the left (authors) to the center (countries) show that the vast majority of authors are from China; the center-to-right (institutions) connections indicate that the Chinese Academy of Medical Sciences & Peking Union Medical College has the strongest Chinese affiliation, followed by Harbin Institute of Technology, Sichuan University, and Guangdong Cardiovascular Institute. Switzerland is mainly associated with University Children's Hospital Zurich, and Singapore is mainly associated with Nanyang Technological University.

### 3.6 Keywords and hotspots

**Figure 7:**
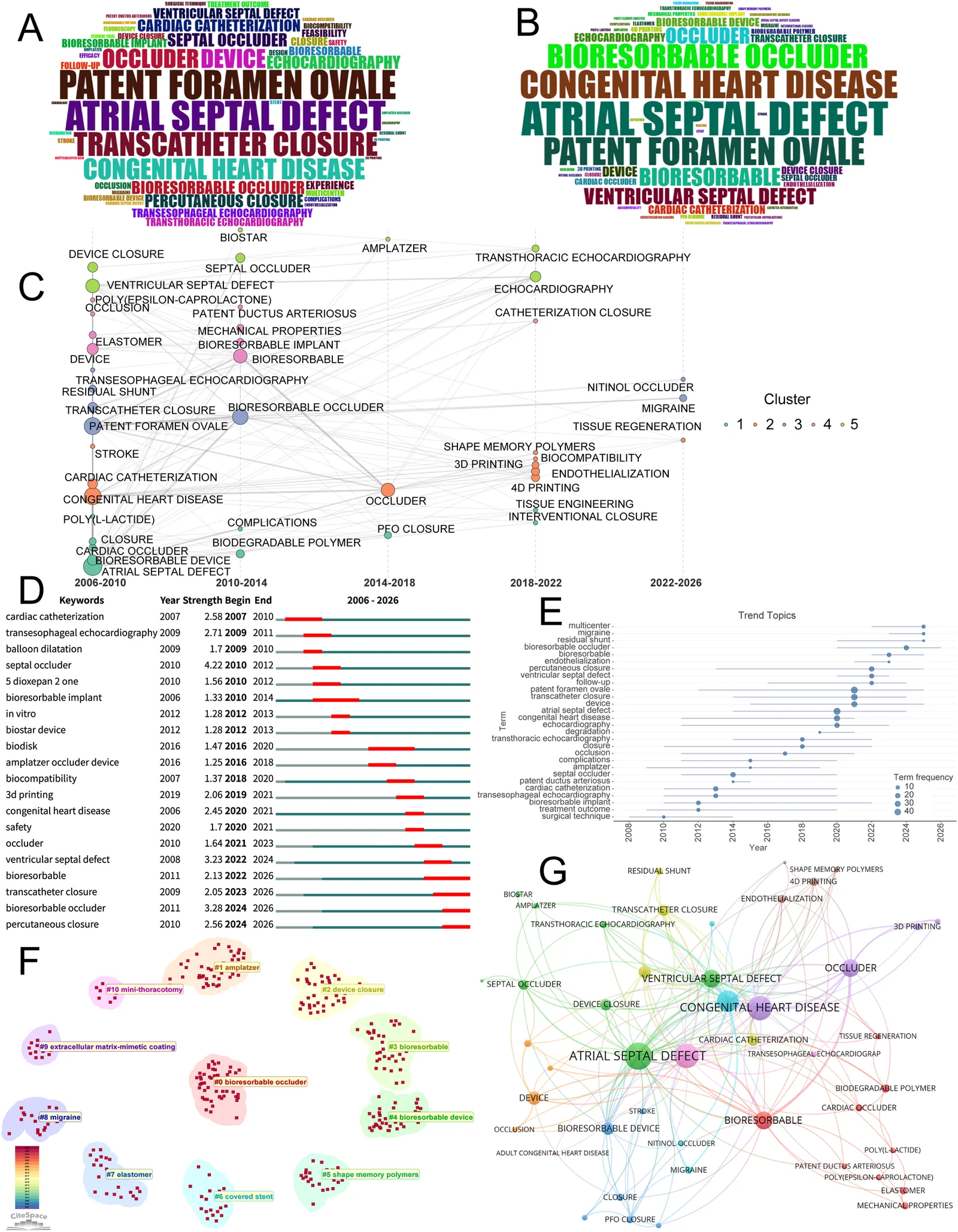
(**A**) Overall keyword-frequency word cloud; (**B**) author-keyword frequency distribution; (**C**) keyword timeline map; (**D**) top 20 keywords with the strongest bursts; (**E**) trend-topic analysis; (**F**) CiteSpace keyword-cluster map; (**G**) VOSviewer keyword co-occurrence network.

Figures 7A,B showed that the principal high-frequency terms included atrial septal defect, patent foramen ovale, transcatheter closure, congenital heart disease and bioresorbable occluder. The keyword co-occurrence network in Figure 7G further demonstrated close connections between clinical defect closure and bioresorbable materials and devices, catheter-based interventions, echocardiographic guidance, advanced manufacturing, tissue integration, and clinical outcomes. Figure 7F contained 342 nodes and 1,436 links and generated 11 clusters, with representative clusters including bioresorbable occluder, device closure, bioresorbable device, shape memory polymers, migraine and extracellular matrix-mimetic coating. Timeline and trend analyses showed that research attention gradually expanded from conventional occluders and catheter-based interventions toward bioresorbable materials, endothelialization, 3D/4D printing, tissue regeneration, and clinical evaluation (Figures 7C,E). Septal occluder showed the strongest keyword burst (strength = 4.22), followed by bioresorbable occluder (3.28) and ventricular septal defect (3.23). Bioresorbable, transcatheter closure, bioresorbable occlude and percutaneous closure remained active burst terms through 2026, indicating sustained attention to the clinical translation of bioresorbable closure technologies (Figure 7D).

### 3.7 Topic modeling

**Figure 8:**
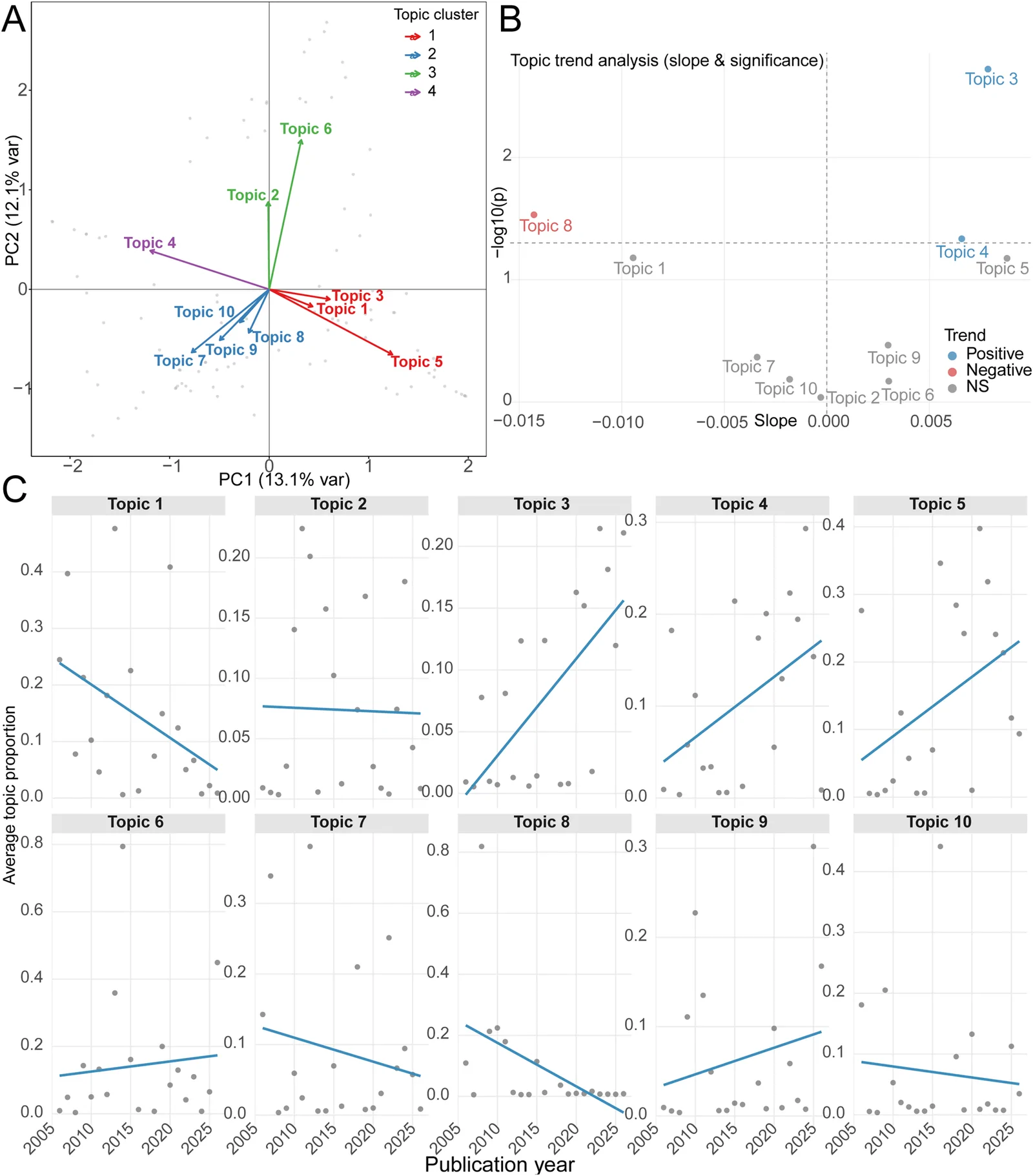
LDA topic modeling analysis results. (**A**) Topic principal component distribution; (**B**) topic trend analysis; (**C**) topic prevalence.

Figure 8 presents the main results of LDA topic modeling. Figure 8A shows the relative positions of the 10 topics in principal component space, reflecting semantic similarities among the topics. Figure 8C indicates that Topic 5—implantation and host response—has the highest prevalence (0.1497), followed by Topic 6—3D/4D printing and personalized occluders (0.1258); Topic 4—preclinical prospects of bioresorbable occluders—and Topic 1—limitations of metal occluders—rank third and fourth, respectively. Figure 8B’s topic trend analysis further reveals each topic's evolution: Topic 3—ultrasound-guided pediatric VSD occlusion—and Topic 4 show significant upward trends (*P* < 0.05), while Topic 8—long-term issues of early ASO devices—shows a significant downward trend (*P* < 0.05); the remaining topics remain stable. These results indicate that scholarly attention is shifting from the limitations of traditional metal occluders toward the preclinical prospects of resorbable occluders, with sustained increases in research interest particularly in ultrasound-guided pediatric interventions and novel resorbable devices (Supplementary Tables 3, 4).

## 4 Discussion

This study provides a field-level bibliometric synthesis of two decades of research on bioresorbable occluders for congenital heart disease. Annual publication output exhibited marked year-to-year fluctuations rather than a sustained pattern of continuous growth, with a local increase during 2009–2012 and renewed, although variable, research activity after 2021. The harmonized keyword analysis further showed that thematic evolution was characterized by gradual expansion rather than rigidly separated stages. Earlier studies emphasized conventional septal occluders, cardiac catheterization, and echocardiography-guided closure. Subsequent research increasingly incorporated bioresorbable implants, degradation, and biocompatibility, whereas more recent studies extended toward bioresorbable occluders, percutaneous and transcatheter closure, endothelialization, advanced manufacturing, and tissue regeneration.

The principal contribution of this analysis is not another device-by-device account, but an integrated view of how the field has developed as a research system. Linking temporal output with geographical participation, collaboration patterns, knowledge foundations, and thematic evolution provides a macroscopic perspective that conventional reviews do not readily capture. This framework helps identify the transition from material proof-of-concept work toward engineering optimization and clinical translation, while also revealing structural gaps, including fragmented international collaboration and limited long-term clinical evidence. These bibliometric findings should guide research prioritization rather than be interpreted as comparative evidence of device safety or efficacy, which requires systematic evidence synthesis and well-designed clinical studies.

The journal co-citation network reveals a key structural feature: the densest co-citation links occur among the blue cluster centered on biomaterials and biomechanics, the red cluster on interventional cardiology, and the yellow cluster at the intersection of materials and interventions. This indicates that the deep integration of material degradation behavior, mechanical performance, and interventional techniques is the primary driving force for sustained development in the field, while fundamental breakthroughs in materials science serve as the wellspring for clinical innovation. Computational simulation methods such as finite element analysis have been widely used to optimize occluder mechanical design, ensuring sufficient radial support while imparting compliance matched to cardiac tissue, thereby reducing mechanical irritation to adjacent conduction systems. From the polymer materials perspective, poly-L-lactic acid (PLLA) and its copolymers have become mainstream choices, but their elastic modulus and shape-recovery capability are far inferior to nitinol, which may result in incomplete shape recovery after device deployment. More critically, the material degradation rate must be precisely matched to the host tissue repair rate. Excessively rapid degradation can provoke inflammatory responses and premature loss of mechanical support, leading to defect recanalization; excessively slow degradation may delay endothelialization and increase thrombotic risk. Current research seeks breakthroughs at the molecular-design level through approaches such as copolymer composition control, topology optimization, and surface functionalization, aiming to synchronize degradation kinetics with the repair process (8, 22). Among these, composite systems combining a PDO framework with a PLLA occlusive membrane have demonstrated good temporal matching in animal experiments; this design concept has subsequently been validated at the clinical-translation level in multicenter randomized controlled trials, reflecting the close symbiosis between basic research and clinical translation (19).

At the level of accumulating clinical evidence, topic modeling shows a marked upward trend for Topic 4 (biodegradable occlusion), while Topic 3 (ultrasound-guided pediatric VSD occlusion) is also increasing; conversely, Topic 8 (long-term issues of early ASO devices) is declining and Topic 7 is stable. This contrast clearly indicates that scholarly attention is shifting from the limitations of traditional metal occluders toward evaluating the prospects of next-generation absorbable solutions. In the field of ASD occlusion, a multicenter randomized noninferiority trial demonstrated that a biodegradable occluder had a noninferior occlusion success rate at 6 months compared with a conventional metal occluder (23). In VSD occlusion, the first multicenter randomized controlled trial of a fully bioresorbable occluder also yielded breakthrough results: the absorbable occluder group had a significantly lower incidence of persistent conduction block than the nitinol group (15). In addition, preclinical studies of a novel fully absorbable ASD occluder based on P(LA/CL) copolymer showed that the device was nearly completely absorbed and replaced by neo-tissue at 18 months (24). For the pediatric population, a single-center phase III clinical trial validated the safety and efficacy of the Absnow fully absorbable ASD occluder in children (25). Meanwhile, an expert consensus on patent foramen ovale has been published, marking the transition of this technology from exploratory use toward standardized application (26). Echocardiography, as the core noninvasive tool for monitoring occluder degradation and endothelial coverage integrity, is having its assessment criteria established (27).

The development of bioresorbable occluders can draw on the experiences of contemporary bioresorbable stents and bioresorbable left atrial appendage occluders. Coronary bioresorbable stents were withdrawn from the market because overly thick strut beams increased thrombosis risk; this warns that bioresorbable occluders must precisely align their degradation time window with mechanical support requirements. Left atrial appendage occluders face a trade-off between the speed of endothelialization and device-related thrombosis, while also demanding a higher standard of sealing completeness. By contrast, occluders for congenital heart disease benefit from stronger peridevice endothelial migration, which favors rapid endothelialization. However, the need to accommodate growth in pediatric patients is a unique challenge seldom encountered by other bioresorbable devices. Bioresorbable occluders can learn degradation kinetic modeling from stent failures, adopt surface modification strategies from left atrial appendage occluders, and, through growth-adaptive design, provide a model for other bioresorbable implants.

However, the limitations of existing clinical evidence cannot be ignored. This study found only 105 included publications; although high-level evidence has increased rapidly in recent years, long-term follow-up data from large multicenter randomized controlled trials remain limited. More importantly, follow-up periods are generally short, mostly 1–2 years, and thus insufficient to fully assess long-term tissue remodeling after complete degradation of absorbable occluders and their lifecycle impact on cardiac function. Existing reviews in the field indicate that degradable implants in pediatric and congenital populations require further long-term study before they can be recommended as routine replacements for permanent devices (18). For children with congenital heart disease—the main indication population—questions remain about whether the neo-tissue at the defect site after occluder degradation will maintain sufficient strength and elasticity throughout growth and development; these issues require long-term follow-up extending into adulthood.

Keyword burst analysis further clarified the changing research frontier. Earlier bursts were associated with cardiac catheterization, transesophageal echocardiography, septal occluders, and early bioresorbable implants, reflecting the initial focus on device-based closure and procedural guidance. More recent and ongoing bursts involved bioresorbable, transcatheter closure, bioresorbable occluder and percutaneous closure, indicating sustained attention to the clinical translation of resorbable closure technologies. In parallel, the co-occurrence and cluster analyses identified endothelialization, extracellular matrix-mimetic coatings, shape-memory polymers, and tissue regeneration as important engineering and biological directions. These developments suggest that occluders are increasingly being conceptualized not only as physical barriers but also as temporary scaffolds capable of supporting tissue integration and repair. At the level of material smart response, near-infrared light-controlled shape-memory occluders have improved the ability of devices to be precisely deployed within complex anatomies (28). At the level of surface functionalization, glycocalyx-mimetic and extracellular matrix-mimetic coatings have been reported to accelerate re-endothelialization and promote tissue healing (12, 29).

In advanced manufacturing, 4D printing technology has been used to fabricate dynamically reconfigurable biomimetic atrial septal defect occluders; *in vitro* and *in vivo* experiments confirmed that integrated designs combining biomimetic configuration, biocompatibility, and biodegradability can eliminate a series of complications associated with traditional occluders, marking a key step in translating 4D-printed occluders from model research to clinical application (30, 31). Building on this, a 4D-printed intelligent PFO occluder has achieved synergistic anticoagulant and pro-endothelialization functions (29, 32). The combination of 4D printing and smart materials has also given rise to a new technological paradigm: dynamic occluders that adapt to growth and development. For pediatric congenital heart disease patients, a core defect of traditional metal occluders is their inability to accommodate cardiac growth. Piayda and colleagues noted that conventional metal occluders, due to continuous compression of surrounding tissues and lack of growth adaptability, may lead to serious long-term complications such as chronic inflammation, myocardial erosion, and delayed atrioventricular conduction block (33). Feins and co-workers provided pioneering experimental evidence by designing a growth-adaptive implant composed of a bioresorbable core and a braided sleeve, demonstrating its feasibility for actively accommodating tissue growth in rat tibia and porcine heart valve models (34). The dynamic morphological adaptability conferred by 4D printing offers the prospect of introducing this design concept into the occluder field, fundamentally addressing the mismatch between fixed-size implants and pediatric growth. Furthermore, tissue-engineered vascular grafts have been shown to remodel and grow with the host into fully functional neovessels, further validating the substantial potential of biodegradable, dynamically adaptive implants in pediatric cardiovascular applications (35).

At the level of national collaboration networks, China is an unequivocal core node, having established extensive cooperative relationships with the United States, Singapore, Germany, Switzerland, France and others, forming a star-shaped collaboration pattern centered on China. However, the centralization of the collaboration network also indicates that there remains structural room for optimization in international academic exchange in this field. On the one hand, collaborations among high-output institutions mostly occur within their own countries or regional clusters; intercontinental projects and truly global multicenter clinical studies remain relatively limited. On the other hand, the proportion of China's internationally coauthored papers is comparatively low and still has substantial room for improvement relative to European and North American countries.

A qualitative examination of selected studies suggests a possible geographical difference in research emphasis. For example, one representative study from China focused on the molecular structural design of degradable materials and the regulation of degradation behavior (8), whereas selected studies from European and North American groups examined device-delivery systems and retrievable delivery strategies (36, 37). However, these examples were derived from a limited number of individual publications rather than a country-stratified bibliometric comparison. They should therefore be interpreted as hypothesis-generating observations rather than evidence of systematic national or regional research tendencies. Future studies based on a larger literature corpus could test this hypothesis through country-stratified keyword, topic, and citation analyses. If confirmed, such differences may provide opportunities for international collaboration linking material innovation, device engineering, and clinical translation.

The paradigm of interventional treatment for congenital heart disease is itself continually evolving. From the early era of transcatheter occlusion guided by x-ray fluoroscopy to the current minimally invasive occlusion performed transthoracically or transesophageally under pure ultrasound guidance, surgical trauma and radiation exposure have steadily decreased. Transesophageal echocardiography can provide clearer images of cardiac structures, is particularly suitable for patients with obesity or significant pulmonary interference, and has become an important adjunct in complex cases; a 2025 review elaborated on its central role in adult interventional treatment of congenital heart disease (38). With deepening research into absorbable occluders, treatment is shifting from device-based occlusion toward tissue regeneration. Within this vision, several frontier directions deserve attention. First, establishing coupled degradation–repair models to enable personalized simulation of degradation programs via multiscale computational modeling. Second, exploring adaptive immune-regulation mechanisms to induce regulatory T cells and establish long-term immune tolerance. Third, integrating local delivery functionalities for drugs and genes to upgrade occluders into comprehensive therapeutic platforms (39). Fourth, deeply integrating artificial intelligence with real-world evidence, using machine-learning models for early warning of postoperative complications. Breakthroughs in these directions will drive the transition of absorbable occluders from passive implants to intelligent, active therapeutic platforms, ultimately realizing the therapeutic ideal of intervention without permanent implantation: temporary scaffolds with permanent healing.

## 5 Limitations

This study has several methodological limitations. First, although both the Web of Science Core Collection and Scopus were searched, some regional journals or non-English literature may still have been missed due to database coverage limitations. Second, literature screening was based on titles, abstracts, and keywords, so subjective influence cannot be entirely excluded. In addition, publication bias may affect the accuracy of trend assessments, and bibliometric methods focus on academic output rather than true clinical efficacy, so interpretation should be integrated with the clinical evidence base. The study's time window ends in early 2026; subsequently emerging high-level evidence may modify the present trend conclusions. Nevertheless, independent analyses and cross-validation across the two databases effectively strengthened the robustness and reliability of the findings. Finally, because the literature corpus was relatively small and national publication output was unevenly distributed, we did not perform a country-stratified thematic comparison. Consequently, systematic differences in research priorities among countries or regions cannot be inferred from the present bibliometric findings.

## 6 Conclusion

From early clinical validation of the partially degradable BioSTAR occluder concept, to randomized controlled trial confirmation of fully degradable occluders represented by MemoSorb in ASD and VSD, and onward to prospective exploration of 4D-printed smart occluders, the development trajectory of absorbable occluders clearly maps an innovation pathway from material substitution to functional reconstruction to active repair. Future research must focus on systematically elucidating the biomechanical coupling mechanisms between degradation kinetics and tissue repair, achieving engineering breakthroughs in integrated intelligent functionality, and establishing risk-assessment systems based on long-term real-world data. In this process, deep integration of materials science, biomechanics, immunology, and clinical medicine will be critical to breaking new ground. More broadly, the successful experience with absorbable occluders is providing a paradigm for the overall advancement of structural heart disease implants: the concepts of temporariness, absorbability, and actively induced tissue regeneration are extending to left atrial appendage occluders, transcatheter valve devices, and even vascular grafts. With sustained interdisciplinary integration and global collaborative innovation, the therapeutic ideal of intervention without permanent implantation and permanent healing is poised to become a reality in this field.

## Data Availability

The datasets presented in this study can be found in online repositories. The names of the repository/repositories and accession number(s) can be found in the article/Supplementary Material.
